# Supplementary material for: Structure, Luminescence and Temperature Detection Capability of [C(NH2)3]M(HCOO)3 (M = Mg2+, Mn2+, Zn2+) Hybrid Organic–Inorganic Formate Perovskites Containing Cr3+ Ions
Source: Sensors (Basel). 2023 Jul 9;23(14):6259. doi: 10.3390/s23146259 (PMC10386541; doi:10.3390/s23146259)
Supplement: Supplementary file 1 [file sensors-23-06259-s001.zip › sensors-2452520-supplementary.pdf]

Supplementary Materials for

Structure, luminescence and temperature detection capability of $[C(NH_2)_3]M(HCOO)_3$ ($M= Mg^{2+}, Mn^{2+}, Zn^{2+}$) hybrid organic-inorganic formate perovskites containing Cr^{3+} ions

Dagmara Stefańska [1*], Adam Kabański [1], Thi Hong Quan Vu [1], Marek Adaszyński [1], and Maciej Ptak [1]

[1] W. Trzebiatowski Institute of Low Temperature and Structure Research of the Polish Academy of Sciences, Wroclaw, Poland;

*Correspondence: d.stefanska@intibs.pl

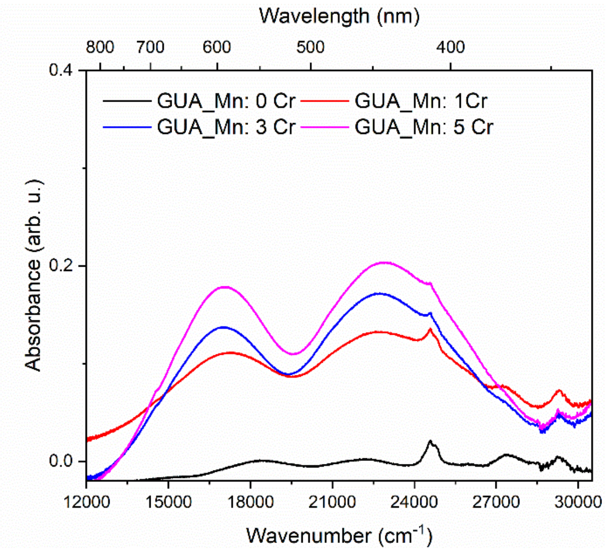

Figure S1. Diffuse reflectance spectra of a series of $[GA]Mn_{1-x}Cr_x(HCOO)_3$ (x = 0, 0.01, 0.03, 0.05) measured at 300 K.

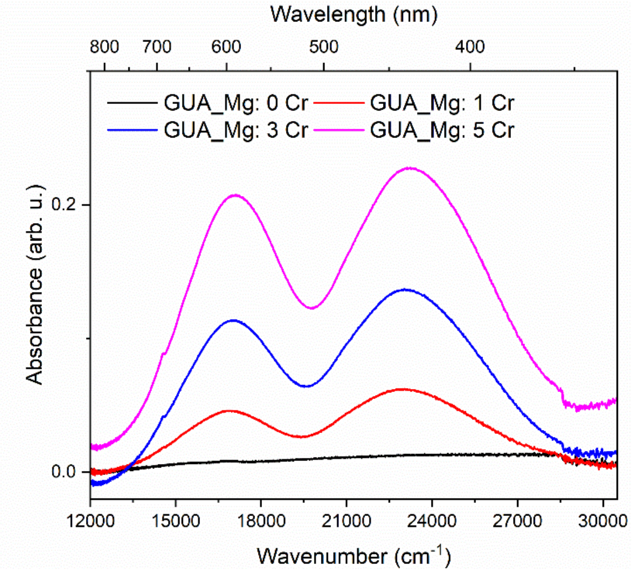

Figure S2. Diffuse reflectance spectra of a series of $[GA]Mg_{1-x}Cr_x(HCOO)_3$ (x = 0, 0.01, 0.03, 0.05) measured at 300 K.

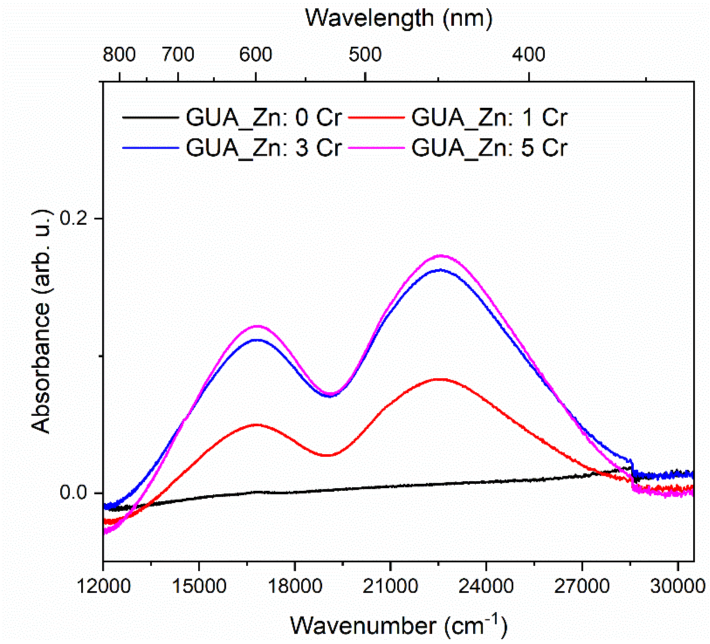

Figure S3. Diffuse reflectance spectra of a series of $[GA]Zn_{1-x}Cr_x(HCOO)_3$ (x = 0, 0.01, 0.03, 0.05) measured at 300 K.

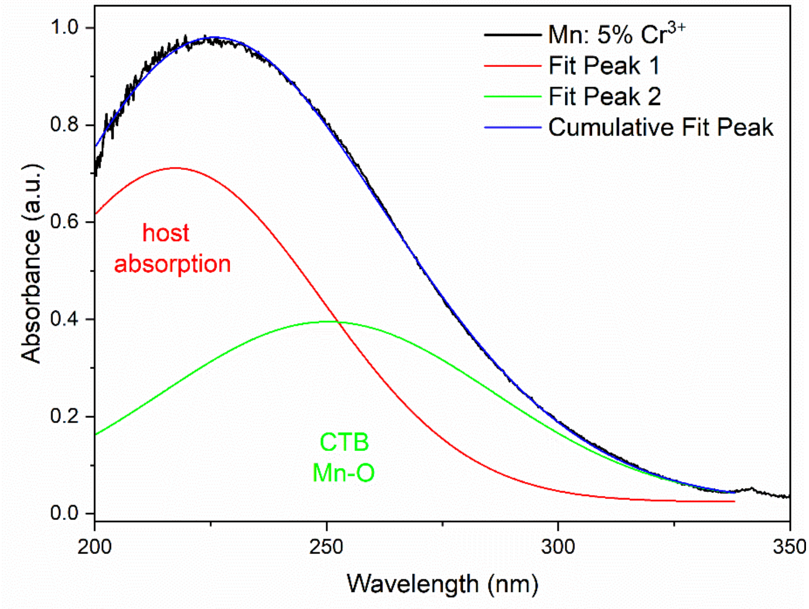

Figure S4. Deconvolution of absorption spectrum of $[GA]Mn_{1-x}Cr_x(HCOO)_3$ (x = 0.05) measured at 300 K.

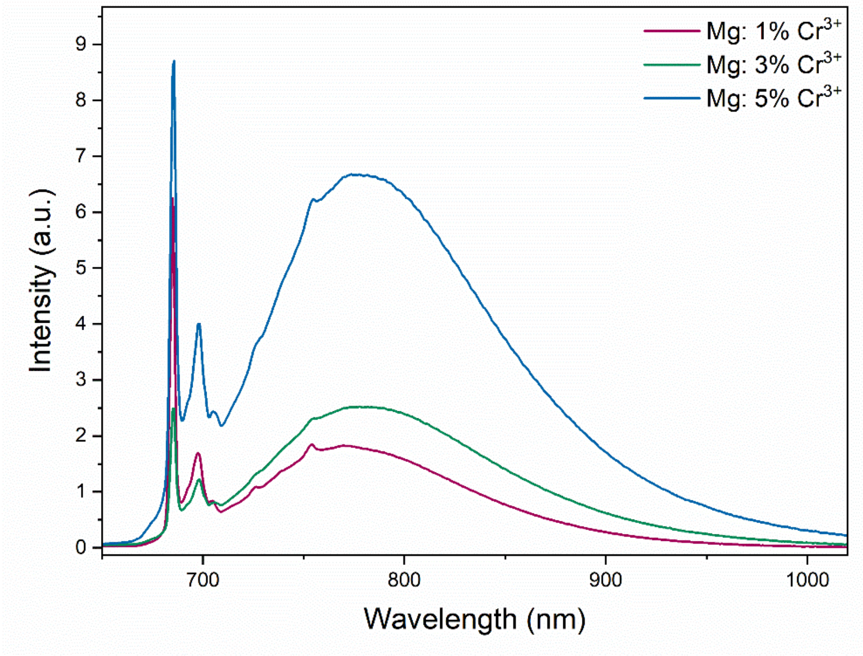

Figure S5. Low-temperature emission spectra of $[GA]Mg_{1-x}Cr_x(HCOO)_3$ (x = 0.01, 0.03, 0.05).

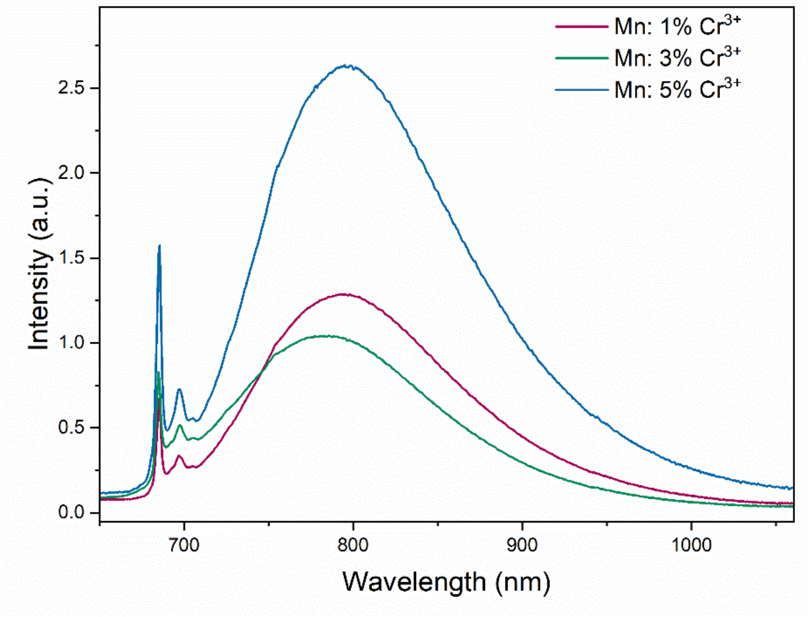

Figure S6. Low-temperature emission spectra of $[GA]Mn_{1-x}Cr_x(HCOO)_3$ (x = 0.01, 0.03, 0.05).

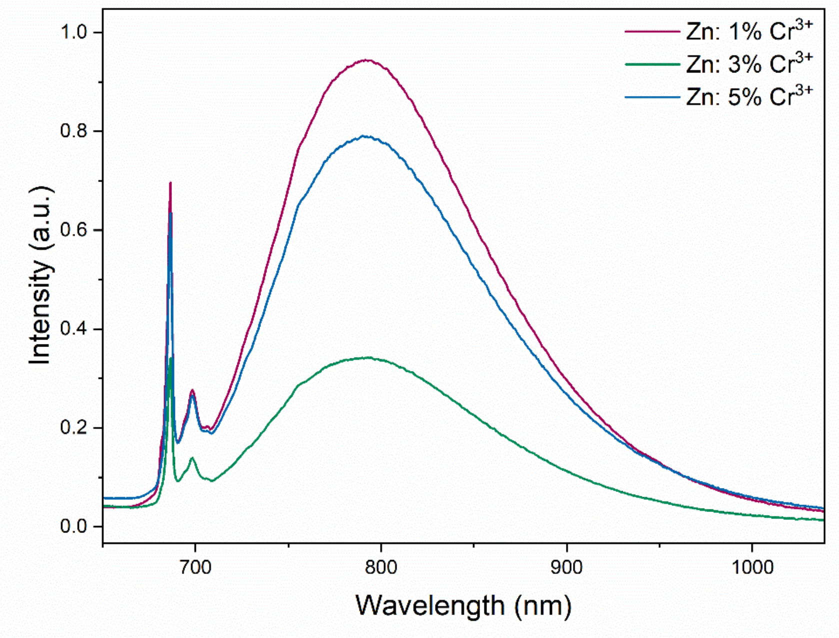

Figure S7. Low-temperature emission spectra of $[GA]Zn_{1-x}Cr_x(HCOO)_3$ (x = 0.01, 0.03, 0.05).

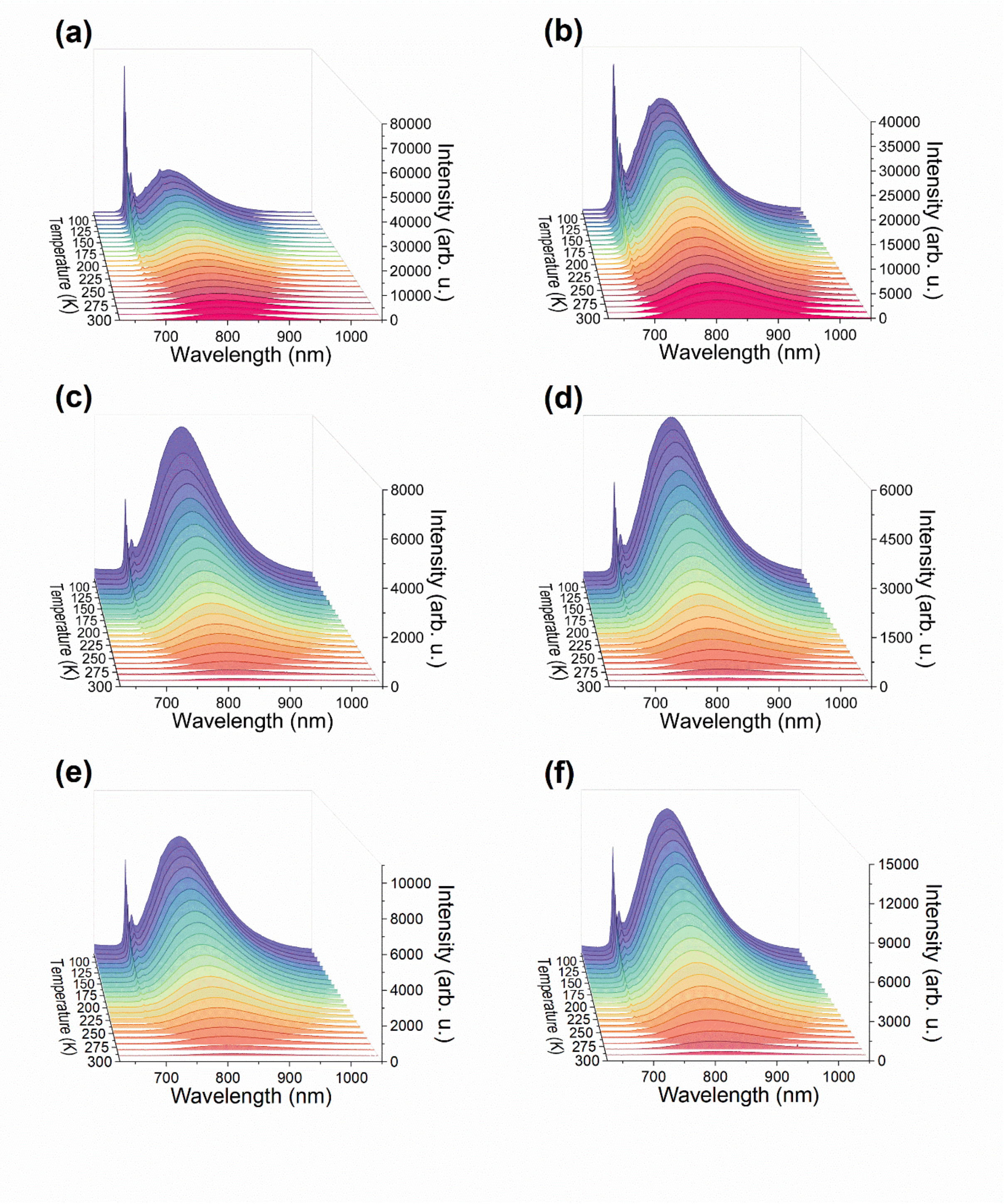

Figure S8. Temperature-dependent emission spectra of $[GA]Mn_{1-x}Cr_x(HCOO)_3$ x= 0.01 (a), $[GA]Mn_{1-x}Cr_x(HCOO)_3$ x= 0.03 (b), $[GA]Mg_{1-x}Cr_x(HCOO)_3$ x= 0.01 (c), $[GA]Mg_{1-x}Cr_x(HCOO)_3$ x= 0.03 (d), $[GA]Zn_{1-x}Cr_x(HCOO)_3$ x= 0.01 (e), and $[GA]Zn_{1-x}Cr_x(HCOO)_3$ x= 0.03 (f) samples.

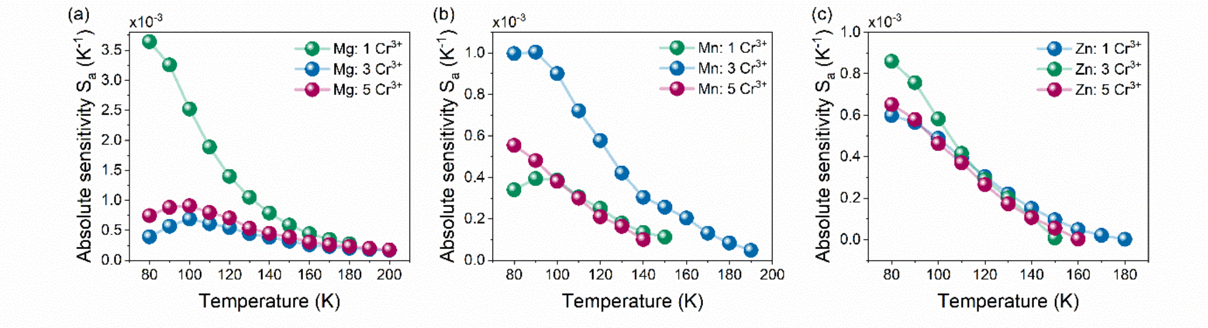

Figure S9 Influence of Cr^{3+} ions concentration on absolute sensitivity (S_a) (a-c) of $[GA]M_{1-x}Cr_x(HCOO)_3$ (M = Mg^{2+}, Mn^{2+}, Zn^{2+}, and x = 0.01, 0.03, 0.05) hybrid perovskites.

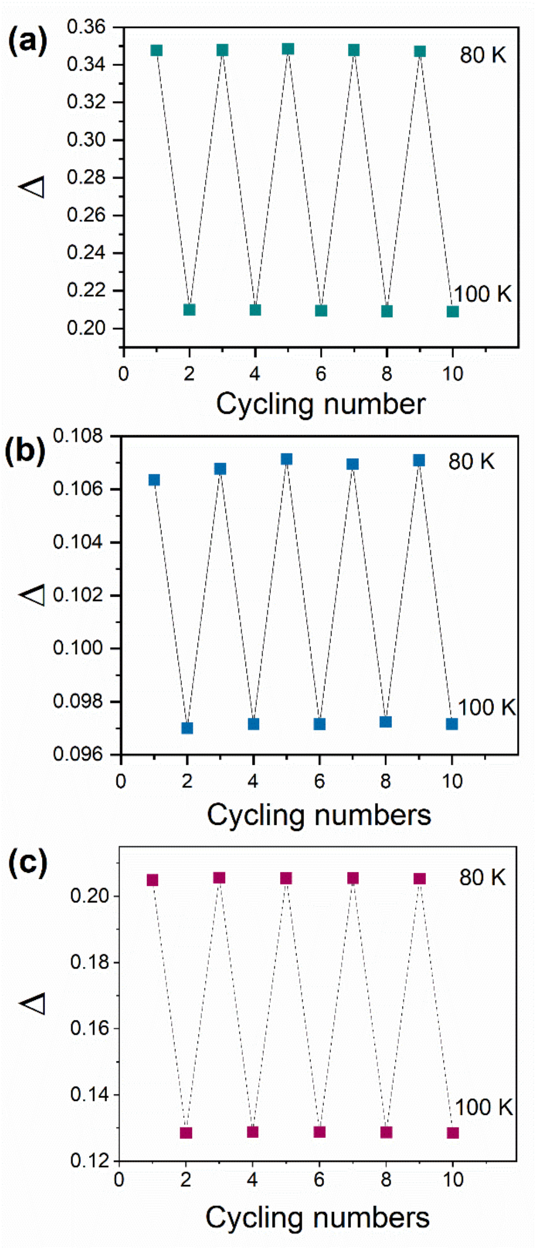

Fig. S10 Repeatability of Δ temperature parameter of I_1/I_2 emission evaluated at 80 K and 100 K during 10 heating/cooling cycles of (a) $[GA]Mg_{1-x}Cr_x(HCOO)_3$ x= 0.01, (b) $[GA]Mn_{1-x}Cr_x(HCOO)_3$ x= 0.03, and (c) $[GA]Zn_{1-x}Cr_x(HCOO)_3$ x= 0.01.

Table S1. Quantities of precursors used for the syntheses of the series of $[GA]Mn_{1-x}Cr_x(HCOO)_3$.

x	HCOOH, m (g)	GA, m (g)	$Mn(ClO_4)_2{\cdot}6H_2O$, m (g)	$CrCl_3.6H_2O$, m (g)
0	0.4	0.7600	0.3618	0
0.01	0.4	0.7600	0.3582	0.0027
0.03	0.4	0.7600	0.3510	0.0080
0.05	0.4	0.7600	0.3437	0.0133

Table S2. Quantities of precursors used for the syntheses of the series of $[GA]Mg_{1-x}Cr_x(HCOO)_3$.

x	HCOOH, m (g)	GA, m (g)	$MgCl_2$, m (g)	$CrCl_3.6H_2O$, m (g)
0	0.4	0.7600	0.0952	0
0.01	0.4	0.7600	0.0943	0.0027
0.03	0.4	0.7600	0.0924	0.0080
0.05	0.4	0.7600	0.0904	0.0133

Table S3. Quantities of precursors used for the syntheses of the series of $[GA]Zn_{1-x}Cr_x(HCOO)_3$.

x	HCOOH, m (g)	GA, m (g)	$ZnCl_2$, m (g)	$CrCl_3.6H_2O$, m (g)
0	0.4	0.7600	0.13630	0
0.01	0.4	0.7600	0.13494	0.0027
0.03	0.4	0.7600	0.13221	0.0080
0.05	0.4	0.7600	0.12949	0.0133

Table S4. Lattice parameters and calculated factors (d_{oct}, average M^{II}–O bond length; V_{oct}, $M^{II}O_6$ octahedral volume; σ^2, bond angle variance; Δ, distortion index) [10.1107/S0021889811038970] for $[GA]Mn(HCOO)_3$ and $[GA]Zn(HCOO)_3$ based on the crystal data published in [10.1002/chem.200901605].

	**$[GA]Mn(HCOO)_3$**	**$[GA]Zn(HCOO)_3$**
a (Å)	8.5211	8.3493
b (Å)	11.9779	11.7276
c (Å)	9.0593	8.9089
V (Å^3)	924.63	872.34
d_{oct} (Å)	2.1611–2.1902	2.0855–2.1267
V_{oct} (Å^3)	13.59	12.22
σ^2 (deg^2)	33.47	30.41
Δ (×10^3)	5.33	7.91

The calculation of the temperature resolution dT has been performed according to the methodology presented by Brites et al. [1]:

$$\delta\mathrm{T} = \frac{1}{\mathrm{S_r}}\frac{\delta\mathrm{FIR}}{\mathrm{FIR}},$$

where S_r is relative sensitivity, and δFIR is the uncertainty of determination of the FIR. The value of the δFIR is a standard deviation of the difference between calculated FIR values and the fitted with the equation describing the thermal quenching of the luminescence [2, 3]:

$$y = \frac{y_0}{1 + A \cdot e^{-\frac{E_a}{k \cdot T}}}$$

where A is a constant value, E_a is the activation energy of thermal quenching, and k is Boltzmann's constant.

[1] C.D.S. Brites, A. Millan, L.D. Carlos, Lanthanides in Luminescent Thermometry, Handbook on the Physics and Chemistry of Rare Earths, 2016, 49, 339-427, https://doi.org/10.1016/bs.hpcre.2016.03.005

[2] M.A. Reshchikov, *Physica Status Solidi a*, 2020, 218, 1, 2000101. https://doi.org/10.1002/pssa.202000101

[3] S. Shionoya, *Photoluminescence* in *Luminescence of Solids*. Springer, Boston, MA. https://doi.org/10.1007/978-1-4615-5361-8_3
